# Twenty weeks of isometric handgrip home training to lower blood pressure in hypertensive older adults: a study protocol for a randomized controlled trial

**DOI:** 10.1186/s13063-018-2441-x

**Published:** 2018-02-09

**Authors:** Martin Grønbech Jørgensen, Jesper Ryg, Mathias Brix Danielsen, Pascal Madeleine, Stig Andersen

**Affiliations:** 1 Department of Geriatric and Internal Medicine, Aalborg University Hospital, Hobrovej 18, 9000 Aalborg, Denmark; 2 Department of Geriatric Medicine, Odense University Hospital, Odense, Denmark; 3 Institute of Clinical Research, University of Southern Denmark, Odense, Denmark; 4 Physical Activity and Human Performance group – SMI, Department of Health Science and Technology, Aalborg University, Aalborg, Denmark; 5 Department of Clinical Medicine, Aalborg University, Aalborg, Denmark

**Keywords:** Isometric handgrip training, Hypertension, Older adults, Home training

## Abstract

**Background:**

Hypertension markedly increases the risk of cardiovascular diseases and overall mortality. Lifestyle modifications, such as increased levels of physical activity, are recommended as the first line of anti-hypertensive treatment. A recent systematic review showed that isometric handgrip (IHG) training was superior to traditional endurance and strength training in lowering resting systolic blood pressure (SBP). The average length of previous IHG training studies is approximately 7.5 weeks with the longest being 10 weeks. Therefore, presently it is unknown if it is possible to further lower blood pressure levels beyond the 10-week mark. Recently, we developed a novel method for monitoring handgrip intensity using a standard Nintendo Wii Board (Wii). The primary aim of this study is to explore the effects of a 20-week IHG home training facilitated by a Wii in hypertensive older adults (50 + years of age) on lowering SBP compared to usual care. Secondary aims are to explore if/when a leveling-off effect on SBP will occur during the 20-week intervention period in the training group and to explore adherence and potential harms related to the IHG home training.

**Methods/design:**

Based on previous evidence, we calculated that 50 hypertensive (SBP between 140 and 179 mmHg), older adults (50 + years of age) are needed to achieve a power of 80% or more. Participants will be randomly assigned to either an intervention >group (IHG home training + hypertension guidelines on lifestyle changes) or to a control group (hypertension guidelines on lifestyle changes). Participants in the intervention group will perform IHG home training (30% of maximum grip strength for a total of 8 min per day per hand) three times a week for 20 weeks. Resting blood pressure and maximal handgrip strength will be obtained by a blinded outcome assessor in both groups at specific time points (baseline, follow-up at 5, 10, 15, and 20 weeks) throughout the study.

**Discussion:**

This assessor-blinded, randomized controlled trial will explore the effect of a 20-week IHG home training intervention on resting blood pressure in hypertensive older adults. In addition, the trial will report adherence and potential harms related to the IHG home training.

**Trial registration:**

ClinicalTrials.gov, ID: NCT03069443. Registered on 3 March 2017.

**Electronic supplementary material:**

The online version of this article (doi:10.1186/s13063-018-2441-x) contains supplementary material, which is available to authorized users.

## Background

Hypertension (HT) is a major public health concern worldwide with an upward trend [1]. HT increases the risk of cardiovascular diseases (e.g., coronary artery disease, stroke, and heart failure) and overall mortality [2, 3]. Both European and US treatment guidelines for primary and secondary prevention of HT recommend non-pharmacological lifestyle modifications, such as increased levels of physical activity, as the first line of anti-hypertensive therapy [4, 5]. In addition, there is class I, level B evidence that 150 min of weekly physical activity offers an alternative that may be used to complement anti-hypertensive medication, although optimal exercise training regime remains unclear [6]. A systematic review from 2013 highlighted the potential of isometric handgrip (IHG) training regimens in reducing systolic blood pressure (SBP) [7]. The meta-analysis from the review showed that IHG training was superior to endurance training and dynamic resistance training in reducing SBP (by − 10.9, − 3.5, and − 1.8 mmHg, respectively) [7]. Another systematic review published in 2014 on IHG training, including isometric leg extension training, reported a more modest mean difference of − 6.77 mmHg in SBP [8]. Lately in 2016, yet another systematic review was published on isometric training (both handgrip and leg extension) and showed an overall reduction in SBP of − 5.2 mmHg [9]. This review, however, was heterogenetic in nature with several outlying studies, which may have undermined the effect of isometric training on blood pressure levels. In addition, some of the studies included in the most recent review were performed on normotensive populations and may have contributed to a reduced effect compared to the systematic review from 2013. Overall, most isometric exercise training studies have followed somewhat the same exercise protocol with an intensity of 30% of maximum voluntary contraction (MVC) in intervals of 2 min per hand/foot (a total of 8 min per hand/foot per day). In addition, the training has been three times per week for an average of 7.5 weeks in duration with the longest reported study being 10 weeks [9]. Most of the studies have been conducted at a hospital, university, or healthcare clinic burdening the participant by having to travel to the study site for assessment and training. Finally, many of the previous studies on IHG training have been relatively small in size (average 28 participants) [9].

Recently, we have shown that handgrip [10] and lower extremity strength [11] can be measured precisely using a standard Nintendo Wii Board (Wii) with an on-screen visual feedback of the force exerted over time (force/velocity curve). As a continuation of this work, we have further developed a novel method that monitors handgrip muscle control and is suitable for IHG home training. Given the development and low-cost nature of the Wii, we are now able to perform home-based IHG training. For the above reasons we intend to conduct a IHG training study, which will be twice as long (20 weeks) as the longest previous study (10 weeks) to explore the effects on specific measures of blood pressure and other relevant outcomes. Our primary aim is to explore the effects of 20 weeks’ IHG home training facilitated by a Wii in hypertensive older adults (50 + years of age) in lowering SBP. Secondary aims are to explore if/when a leveling-off effect on SBP will occur during the 20-week intervention period in the training group and to explore adherence and potential harms related to the IHG home training. The study will compare IHG home training with a control group in a randomized, assessor-blinded design. Thus, we hypothesize (1) that 20 weeks of IHG home training will significantly reduce SBP in hypertensive older men and women compared to usual care, (2) that a leveling-off effect on SBP will occur during the 20 weeks of IHG training, and (3) that a minimum of 50% of participants will complete at least 50% of their training sessions (30 sessions).

## Methods/design

### Design

**Fig. 1:**
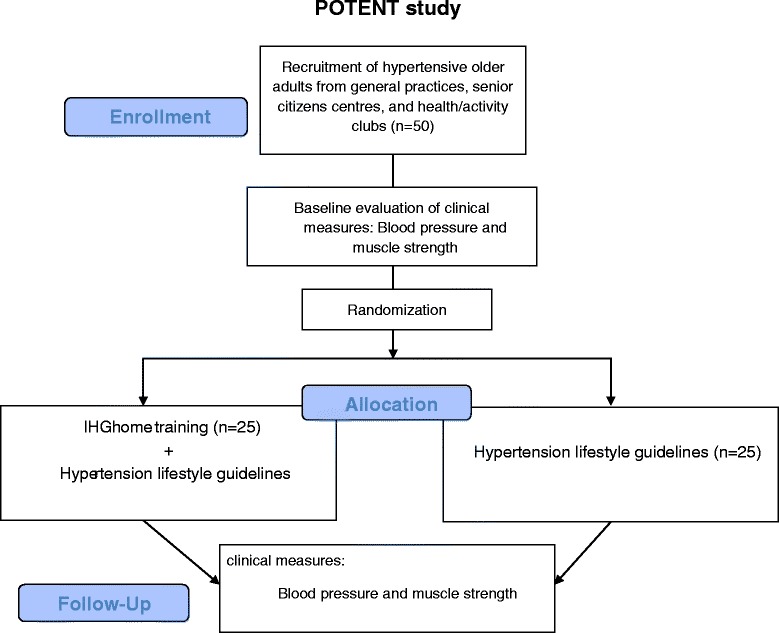
Flowchart of the POTENT study. *IHG* isometric handgrip training

The design of the study will be a randomized controlled, outcome-assessor-blinded superiority trial with participants randomly assigned to either an intervention group (IHG home training + hypertension guidelines on lifestyle changes) or to a control group (hypertension guidelines on lifestyle changes). The length of the study will be 20 weeks and the IHG training will be performed in the participant’s own home. Randomization will be made using a computer-generated block randomization with 1:1 allocation between the intervention group and the usual-care group (see Fig. 1). The protocol follows the Standard Protocol Items: Recommendations for Interventional Trials (SPIRIT) 2013 guidelines Additional file 1 [12].

### Participants and setting

The study will be conducted at one study site: Aalborg University Hospital, Department of Geriatric and Internal Medicine, Aalborg, Denmark. Participants will, therefore, primarily consist of individuals living in an urban environment. Using the criteria below, we attempt to target a study population which has a 15–20% or medium absolute risk of stroke or myocardial infarction within a 10-year period according to the Danish Hypertension Society [13]. Written informed consent will be obtained from each participant.

#### Inclusion criteria

Participants eligible for the POTENT study must comply with all of the following criteria prior to randomization:

1. Age 50 + years old
2. Have a resting SBP between 135 and 179 mmHg and either taking no actual anti-hypertensive medicine or taking actual anti-hypertensive medicine with no treatment change within the last 4 months prior to enrollment

#### Exclusion criteria

1. Manifest cardiovascular disease (cerebrovascular disease, heart failure, chronic kidney disease, peripheral vascular disease, or advanced retinopathy (fundus hypertonicus grades III–IV))
2. Diabetes (any type)
3. More than three blood-pressure-regulating agents
4. Physical limitation preventing IHG training (e.g., missing an arm or having a musculoskeletal disorder)
5. Severe arthritis in the hand, or carpal tunnel syndrome
6. A resting SBP ≥ 180 mmHg (will be advised to consult a physician)

### Sample size

Based on previous evidence [14], and using a more conservative approach, we carried out an a priori power analysis assuming a 7-mmHg difference in the ∆SBP between the IHG training and the usual-care group (favoring the IHG training group) after 20 weeks of training. In addition, we assumed the standard deviation of the IHG training and usual-care group to be 8 and 9 mmHg, respectively. We further choose a power of 80% and an alpha level of 5% with equal group sizes. This resulted in a total sample size of 40 participants with 20 in each group. Assuming a 25% loss to follow-up, we would need to recruit 25 participants in each group.

### Recruitment and randomization procedure

Participants will be recruited consecutively by their local physician, through bulletin, newspaper advertisement, local senior citizens’ centers, and health/activity clubs. Eligible subjects, who agree to participate, will be asked to provide an informed written consent, undergo baseline assessment, and will thereafter be randomized to one of the two arms: (1) IHG training or (2) usual care. Participants will randomly be assigned by computer-generated random numbers in permuted blocks to attend either arm for 20 weeks. The randomization will be stratified according to gender (male/female). A third party not involved in the day-to-day running of the study will perform group allocation and will notify the study coordinator by e-mail to ensure concealed allocation.

### IHG training group and usual-care group

**Fig. 2:**
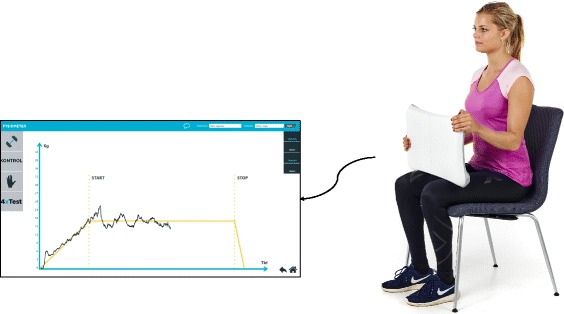
Illustration of the target line and the “live” applied muscle force participants will be seeing during an isometric handgrip (IHG) training session

Participants of the intervention group will follow the home-based IHG training protocol for 20 weeks. The IHG training consists of isometric contractions applied with the hands on a Wii. The Wii will be connected to a standard PC to monitor the level of the applied force using the FysioMeter software (Brønderslev, Denmark). The IHG training will be structured with four sets of 2-min contractions for each hand, 3 days per week for 20 weeks. The training volumes will this way sum up to a total of 480 min for each hand in the study period. The level of IHG training will be set to 30% MVC in line with previous IHG training trials [7, 9]. During training sessions, participants will be seated on a standard chair with the non-working hand holding on to the Wii at the middle of the board, while the working hand will grab the corner of the Wii (see Fig. 2). Participants will alternate between the left and right hand with a 1-min resting period between changing hands. The software displays the target force level (30% MVC) on-screen as a horizontal line (yellow line, see Fig. 2) and participants will apply the required force level on the Wii according to this line (black line, see Fig. 2). Participants will be asked to do the IHG training every other weekday (i.e., Monday, Wednesday, and Friday) allowing for restitution between training days.

Participants will be instructed to breathe at a normal rhythm and depth during IHG training in order to avoid Valsalva maneuvers. To ensure that participants work at 30% MVC throughout the trial, the 30% MVC will be adjusted in the intervention period (after 5, 10, and 15 weeks) according to a previously described method [10].

The usual-care group will have the same number of hospital visits for measurements of blood pressure and MVC as the intervention group in order to ensure similar attention provided by the healthcare professionals.

### Assessment protocol

**Fig. 3:**
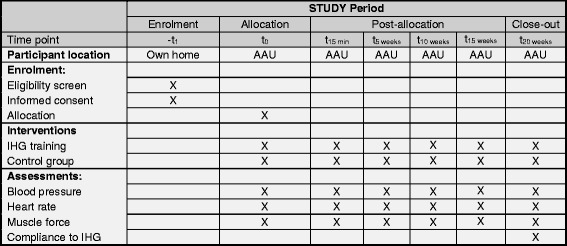
Schedule of enrollment, interventions, and assessments

Prior to the randomization process, participants will undergo demographic and physiological baseline assessments by an experienced assessor. Demographic measures will include: gender, age, height, Body Mass Index (BMI), level of physical activity using the International Physical Activity Questionnaire (IPAQ) [15], co-morbidities, alcohol intake, smoking status, and number and type of medical drugs. Participants will be given advice on lifestyle changes according to the national guidelines including, but not limited to, the DASH Diet [16], reduced alcohol intake, cessation or reduction of smoking, and advice on an active lifestyle [13]. Following randomization all participants will return to Aalborg University Hospital at weeks 5, 10, 15, and 20 for follow-up assessments (see Fig. 3 for an overview based on the SPIRIT Figure) in a controlled environment. At the follow-up measures, participants will be specifically asked not to mention nor comment on which group they have been assigned to in order to maintain allocation concealment to the outcome assessor. With respect to physiological measures, these will be assessed approximately at the same time of day as the baseline measures to avoid variability due to circadian rhythms or medication intake cycle. In addition, participants will be instructed via telephone by the study coordinator to refrain from vigorous exercise and alcohol for 24 h prior to all measurements. Moreover, they will be instructed to fast and refrain from caffeine for 4 h prior to all measurements. Further, they will be instructed to refrain from smoking and intake of anti-hypertensive medicine up to 30 min prior to all measurements. In addition, the assessor will ask if the participants complied with these instructions. The noise level and room temperature (20–23 °C) will be controlled by the assessor prior to the assessments. Finally, participants will be asked to empty their bladder, if possible, before tests by the assessor.

### Outcome measures

#### Primary outcome measure

The primary endpoint in the study is SBP, which will be measured using a standard hospital electrical sphygmomanometer (Omron Comfort M6 AC). After the participants have been relaxing for 5–10 min the SBP measurements are performed on the left arm while it is resting on a table. Participants will be instructed to sit with their legs parallel and to stay quiet during the measurements. SBP will be measured three times in the left arm with rest periods of 1 min between measurements. The average of the three measures at baseline will be used for analysis. For the home SBP recordings the participant will be asked to follow the same procedures (and use the same device) as described above and perform recordings on the same weekday and timeframe as their baseline tests at the hospital. The home measures will be performed prior to commencement of the IHG training on the relevant day.

#### Secondary outcome measures

Diastolic blood pressure (DBP) and heart rate (HR) will be measured using the same procedure as described above for SBP. Exerted force (maximum voluntary force, rate of force development, and force variability) will be measured in both hands using the same procedure as described in detail previously [10, 17, 18]. Further, at the end of each training session participants are asked to self-report pain intensity using a Visual Analog Scale (VAS) [19] for each hand. The VAS reports values between 0 and 10 with 0 corresponding to no pain and 10 to maximum pain. Finally, compliance of the IHG training group will be monitored by a computer-generated report every week during the intervention period.

### Data analysis

Study data will be collected and managed using REDCap electronic data capture tools hosted at Aalborg University Hospital [20]. REDCap (Research Electronic Data Capture) is a secure web-based application designed to support data capture for research studies, providing (1) an intuitive interface for validated data entry; (2) audit trails for tracking data manipulation and export procedures; (3) automated export procedures for seamless data downloads to common statistical packages; and (4) procedures for importing data from external sources. In addition, the study has been reported to the Danish Data Protection authorities.

Statistical analysis of data will be performed using SPSS (Version 22, IBM Corporation, Armonk, NY, USA). Prior to all statistical analysis the dataset will be explored according to parametric test assumptions, and, if violated, an appropriate transformation or statistical method will be applied. Further, statistical analysis will be conducted according to the intention-to-treat principal. However, a sensitivity analysis using the per-protocol principal will also be performed. This will only include participants who have completed minimum 50% of their training sessions (480 min). The between-group difference in the ∆ change from baseline to the 20-week follow-up mark for the primary endpoint will be analyzed using a linear mixed-effects model. Secondary outcomes will also be analyzed using a linear mixed-effects model. Besides these primary analyses a number of sub-analysis will also be performed. Exploring difference in effect of training whether on blood pressure regulating medicine or not, age, gender, and physical activity level (IPAQ). Finally, an analysis of variance (ANOVA) model will be applied after 10 weeks of training to explore if there exists a leveling-off effect on SBP in the IHG group.

### Safety considerations, adverse events, and termination of study

Throughout the trial period, participants are encouraged to contact the study coordinator if they experience any side effects from the IHG training. In addition, during the 5-, 10-, 15-, and 20-week follow-ups all participants will be asked if they have any discomfort or encountered any harms by being part of the study. Muscle soreness may occur during and after the training sessions. This is also seen following traditional strength training and is expected to diminish within 1–2 days [21]. Furthermore, blood pressure might rise slightly during training as seen in running or swimming. Participants will be removed from the study if they start/adjust/terminate anti-hypertensive drugs during the trial. Likewise, if they die or emigrate they will be removed from the study.

## Discussion

The aim of this randomized control trial is to explore whether 20 weeks of IHG home training on older adults (50 + years of age) with hypertension can reduce SBP compared to usual care. In order to increase the clinical value of this study, our inclusion and exclusions criteria are selected to target patients where lifestyle changes are recommended by national guidelines as first line therapy before initiation of medical treatment.

This study adds further knowledge to the current academic literature in several aspects. Firstly, the duration of the intervention in this study will be 20 weeks compared to the average durations of 7.5 weeks seen in previous studies with the longest reported study of 10 weeks. Secondly, with a longer study duration and measurements every 5 weeks, this study should be able to reveal when, and if, a possible leveling-off effect on measures of blood pressure occurs. Thirdly, the present study will extend the size of previous IHG training studies as former studies have included smaller sample sizes (on average approximately 28 participants) [9]. Fourthly, this is the first study to explore the effects on resting blood pressure using a standard Wii for IHG training. Finally, in the present study participants will perform home-based IHG training. Previous studies report IHG training taking place in either a hospital or university setting burdening participants with traveling time and changes in environment and potentially affecting their adherence to the training program. If the home-based IHG training is potent in lowering resting blood pressure this type of setup with home training could be attractive to many patient populations.

### Trial status

Recruitment of participant will be initiate approximately 1 month from now.

## Additional file

Additional file 1: SPIRIT 2013 Checklist: recommended items to address in a clinical trial protocol and related documents. (DOC 122 kb)

## Abbreviations

HT: Hypertension
IHG: Isometric handgrip
SBP: Systolic blood pressure
VAS: Visual Analog Scale
Wii: Nintendo Wii Board
